# Basal Ganglia Involvement in Pediatric *Mycoplasma pneumoniae* Meningoencephalitis: Two Cases and a Literature Review

**DOI:** 10.3390/epidemiologia6040062

**Published:** 2025-10-10

**Authors:** Dominik Ljubas, Luka Švitek, Lorna Stemberger Marić, Nina Krajcar, Maja Vrdoljak Pažur, Ana Tripalo Batoš, Srđan Roglić, Goran Tešović

**Affiliations:** 1 Department of Pediatric Infectious Diseases, University Hospital for Infectious Diseases “Dr. Fran Mihaljević”, 10 000 Zagreb, Croatia; dljubas@bfm.hr (D.L.); sroglic@bfm.hr (S.R.); gtesovic@bfm.hr (G.T.); 2 Department of Infectology and Dermatovenerology, Faculty of Medicine Osijek, Josip Juraj Strossmayer University of Osijek, 31 000 Osijek, Croatia; 3 Clinic for Infectious Diseases, University Hospital Centre Osijek, 31 000 Osijek, Croatia; 4 Department of Infectious Diseases, School of Medicine, University of Zagreb, 10 000 Zagreb, Croatia; 5 Department of Paediatric Radiology, Children’s Hospital Zagreb, 10 000 Zagreb, Croatia; abatosh@gmail.com

**Keywords:** encephalitis, basal ganglia, magnetic resonance imaging, *Mycoplasma pneumoniae*

## Abstract

Background: *Mycoplasma pneumoniae* is a common cause of respiratory tract infections in children, but neurological complications, including encephalitis, are increasingly recognized. Basal ganglia involvement is rare, and a poorly characterized feature of meningoencephalitis, with clinical consequences being inconclusive. Methods: We report two pediatric cases of *Mycoplasma pneumoniae*-related meningoencephalitis with bilateral basal ganglia lesions seen on MRI. A literature review was conducted using PubMed, Scopus, and Web of Science to identify reports of *M. pneumoniae*-related meningoencephalitis in children, and related MRI findings. Results: Both patients (12-year-old male and 14-year-old female) presented with acute meningoencephalitis syndrome and had marked mononuclear pleocytosis. In both patients *M. pneumoniae* was confirmed with serological assay from serum sample, while in one patient *M. pneumoniae* was also confirmed by PCR from pharyngeal swab. Both exhibited bilateral basal ganglia lesions, with complete regression observed during follow-up. Treatment with corticosteroids led to full recovery in both cases. After a literature search, a total of 21 patients had basal ganglia involvement reported. Conclusions: Literature suggests variable MRI findings in pediatric *M. pneumoniae* encephalitis, with basal ganglia involvement being uncommon and rarely reported, especially among older children. While diagnostic challenges related to extrapulmonary manifestations of the infection persist, basal ganglia involvement could aid in diagnosis, especially in older children presenting with meningoencephalitis along with pronounced pleocytosis when respiratory symptoms are absent or mild.

## 1. Introduction

*Mycoplasma pneumoniae* (Mp) is a frequent cause of acute upper and lower respiratory tract infections (RTIs), including community-acquired pneumonia (CAP) among children over 5 years old and adolescents [1]. Along with RTI, extrapulmonary manifestations of the infection have also come to attention in recent years, with the incidence ranging from 25 to 35% [2].

The central nervous system (CNS) is one of the most commonly affected sites in cases of extrapulmonary involvement among pediatric patients [3,4]. Various neurological manifestations attributed to Mp have been described in the literature so far, including meningoencephalitis (ME), aseptic meningitis, myelitis, cerebellitis, Guillain-Barré syndrome, and acute disseminated encephalomyelitis (ADEM) [5,6]. Among these, acute encephalitis syndrome is the most common neurological manifestation of the infection [7]. The overall prevalence of Mp encephalitis (MPE) among pediatric encephalitis cases is estimated to be 5 to 10%, although rates as high as 30% have been reported [8,9]. The recent increase in Mp infection rates following the decline of SARS-CoV-2 pandemic might contribute to an upsurge of extrapulmonary manifestations of the disease, including CNS manifestations [10]. However, diagnosing Mp-related CNS disease, especially MPE, remains challenging due to limited sensitivity and specificity of currently available diagnostic methods, including serological assays and targeted molecular testing [3,11]. Moreover, no clinical sign is sufficient to differentiate MPE from other encephalitides. Despite CNS involvement being increasingly recognized, characteristic magnetic resonance imaging (MRI) features remain poorly defined, and there is still insufficient evidence of potentially distinguishing imaging features [9]. According to the literature, basal ganglia involvement has been occasionally documented in a small number of pediatric patients who presented with findings consistent with MPE [12,13,14].

In view of this evidence, we present two patients with confirmed Mp infection who were treated one year apart at our hospital due to acute ME syndrome, both of whom demonstrated bilateral hyperintense lesions of basal ganglia on MRI, along with prominent pleocytosis and a mild clinical form of ME. In addition to case presentations, we performed a narrative literature review of MPE, with emphasis on MRI features and basal ganglia involvement to summarize current evidence regarding clinical aspects, diagnosis, management and basal ganglia involvement in pediatric MPE.

## 2. Materials and Methods

We present two pediatric cases of MPE treated at the University Hospital for Infectious Diseases “Dr. Fran Mihaljević” in Zagreb, Croatia, between June 2024 and May 2025. In addition, we conducted a narrative literature review to summarize the current evidence on MPE, neuroradiological involvement, with emphasis on basal ganglia involvement, diagnosis, and management.

Relevant publications were identified through targeted searches in PubMed, Scopus, and Web of Science (January 1989–June 2025) using combinations of terms related to *M. pneumoniae*, neurological manifestations, encephalitis subtypes, and basal ganglia. We also screened reference lists of key articles to identify additional reports. The search was limited to human studies involving pediatric patients (0–18 years) and to articles published in English. Studies were eligible if they reported cases of MPE with microbiological confirmation of *M. pneumoniae* infection (via PCR, culture, or serology), and included MRI data, as our aim was to present and summarize neuroradiological characteristics of MPE with a focus on basal ganglia involvement. Articles involving only adult patients, and those lacking microbiological confirmation were not included in the review. Following the selection process, a total of 28 articles were selected. However, potential bias should be acknowledged, as some studies did not provide detailed methodological descriptions of the diagnostic procedures performed to exclude alternative causes of ME syndrome.

Relevant data were extracted from each included article, including the number of pediatric patients, neuroimaging findings, cerebrospinal fluid analysis, microbiological evidence of infection, and treatment. Due to heterogeneity and predominance of case reports and small series, no meta-analysis was performed.

## 3. Case Presentations

Patient 1 and Patient 2 were treated in May 2025 and June 2024, respectively. In both patients, comprehensive diagnostic work-up was done to establish etiological diagnosis of acute ME syndrome, including whole blood count, serum analysis, chest X-ray, and neuroradiological work-up. Lumbar puncture (LP) was performed to assess CSF cell count, blood-brain barrier (BBB) dysfunction and CSF protein levels. We performed targeted serological and polymerase chain reaction (PCR) testing, as well as PCR panel testing (BIOFIRE^®^ FILMARRAY^®^ Meningitis/Encephalitis Panel, bioMérieux, Marcy-l’Étoile, France) for possible infectious agents from serum and CSF samples, as appropriate. In addition, targeted and multiplex PCR testing (Respiratory-well 16, AusDiagnostics, Sydney, Australia) for respiratory pathogens was performed from nasopharyngeal and throat swabs, along with collecting other appropriate blood and stool specimens for microbiological analysis. Serum IgM and IgG antibodies to Mp were measured using a chemiluminescent immunoassay (CLIA) on the LIAISON^®^ XL analyzer (DiaSorin S.p.A., Saluggia, Italy). IgM results were expressed as index values, with results <10 considered negative and ≥10 considered positive. IgG concentrations were expressed in arbitrary units per milliliter (AU/mL), with values <10 AU/mL considered negative and ≥10 AU/mL considered positive. Furthermore, laboratory investigations (thyroid function tests, immune disorders panel, Autoimmune encephalitis panel, EUROIMMUN, Lübeck, Germany) were also performed to exclude non-infectious etiologies, including autoimmune, metabolic, and inflammatory causes of encephalitis.

After extensive diagnostic work-up, the only relevant etiological agent in both patients was Mp, confirmed by serological testing in both patients, along with positive PCR testing from respiratory specimens in one patient. In both cases, chest X-ray revealed no concomitant pneumonia. Mp PCR testing and serology from CSF samples in both patients were performed, which came back negative.

Both of our patients were treated by non-pulse doses of corticosteroids, along with supportive measures, and achieved a fast and full recovery.

Written informed consent for inclusion in this review article was obtained from the legal guardians of the patients.

### 3.1. Patient 1

Patient 1, a previously healthy 12-year-old male, presented with diffuse headache, fever of 38.5 °C, vomiting, photophobia, confusion, and dysarthria. He was admitted to our department on the third day of his illness. Upon admission, a LP and brain MRI were performed (Table 1). LP revealed marked pleocytosis, predominantly mononuclear, with mildly elevated CSF protein levels. The results, combined with the clinical presentation, were consistent with a diagnosis of acute ME syndrome.

Approximately two weeks prior to the current disease, the patient experienced a single one-day episode of fever with cough, nasal congestion, and headache. He was treated with azithromycin for 3 days, which was prescribed by his family physician. His symptoms improved following antimicrobial therapy. Due to nonspecific respiratory symptoms and headache prior to current illness, diagnostic evaluation for enteroviral and Mp infection was performed. PCR from a throat swab and CSF was negative for both agents, as well as enteroviral stool isolation, but serum serological testing was suggestive of a recent Mp infection (IgM > 27 (index) and IgG 57 AU/mL).

**Figure 1:**
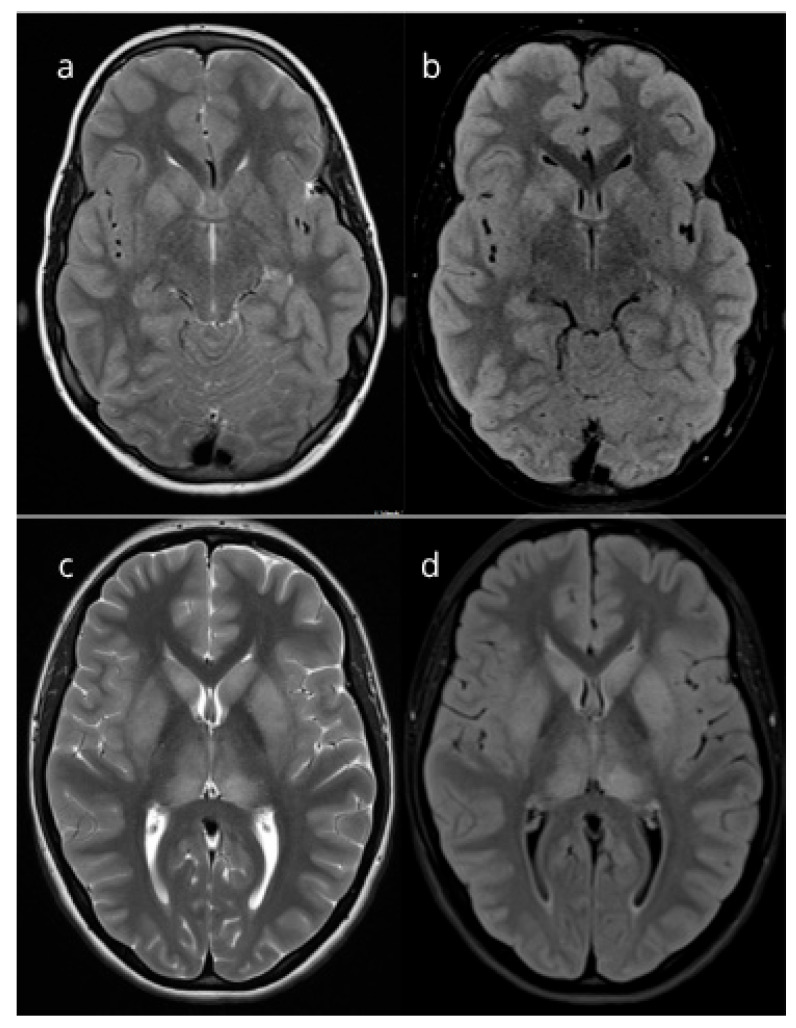
Magnetic resonance imaging (MRI) findings in patients. Axial T2-weighted (**a**) and axial FLAIR (**b**) MRI images of Patient 1 show bilateral asymmetrical hyperintensities in the head of the caudate nuclei and the right putamen, suggestive of encephalitis. Axial T2-weighted (**c**) and axial FLAIR (**d**) MRI images of Patient 2 demonstrate bilateral symmetrical hyperintensities in the basal ganglia and thalami, also suggestive of encephalitis.

In addition, an extensive evaluation for other possible etiologies of encephalitis was conducted, both infectious and non-infectious. This included CSF cultures, PCR testing of CSF sample for herpes (HSV) and varicella viruses (VZV), enteroviruses (EVs), other viruses and bacterial agents, as well as serum and CSF serologies, RT-PCR for arboviral infections and borreliosis, and autoimmune encephalitis panels. All performed tests returned negative. Neuroradiological examination with MRI was performed on the third day of current illness (16 days after the initial respiratory illness) and showed basal ganglia lesions (Figure 1).

Empirical acyclovir therapy was initiated and continued until RT-PCR results from CSF for HSV-1, HSV-2, and VZV returned negative. Since the clinical course was indicative of an immunological phase of the disease and given the fact that PCR results for common causes of aseptic meningoencephalitis were negative, along with negative targeted PCR testing for Mp, corticosteroid therapy was administered (Table 1) Initially, he was treated with methylprednisolone (2.5 mg/kg) for 5 days, followed by a 5-week taper-off with prednisone, starting at 60 mg daily then reducing the daily dose weekly to 45 mg, 30 mg, 20 mg, 15 mg, and finally 10 mg.

At the time of discharge, the patient was fully recovered, with no residual neurological deficits. Clinical and neuroradiologic follow-up was performed three months later, with MRI scans showing regression of the lesions.

### 3.2. Patient 2

Patient 2, a previously healthy 14-year-old female presented with frontal headache, fever of 39.5 °C, vomiting, drowsiness and photophobia. She was admitted to our department on the second day of her illness. Physical examination revealed positive meningeal signs, mild lethargy, and slightly blurred vision, with no other abnormalities. One week prior to the current illness, the patient had experienced symptoms of common cold without fever.

The same laboratory and microbiological investigations as in Patient 1 were performed to detect possible infectious or non-infectious cause of ME. Serological testing for Mp was consistent with a recent infection, showing IgM > 27 (index) and IgG of 75 AU/mL suggesting that minor respiratory complaints one week prior might be associated with the infection. In addition, PCR from a throat swab was positive for Mp, while all other performed tests returned negative, including targeted *M. pneumoniae* PCR from CSF sample. Her chest X-ray was normal. MRI scan performed on the seventh day of her current illness (14 days after the episode of common cold) showed bilateral symmetrical lesions of the basal ganglia (Figure 1).

In addition to empirical acyclovir therapy (which was discontinued following negative CSF PCR results for HSV-1, HSV-2, and VZV), and corticosteroid treatment (Table 1). She was treated with methylprednisolone (2.5 mg/kg) for 5 days, followed by a 4-week taper-off with prednisone, starting with 60 mg daily then reducing the daily dose weekly to 40 mg, 25 mg, and finally 15 mg. In addition, we decided to administer a 5-day course of azithromycin due to a positive Mp PCR result from a pharyngeal swab. Azithromycin was administered despite it being more likely that the positive PCR reflected a recent, self-limiting respiratory tract infection, while the current disease phase appeared immune-mediated, as supported by the medical history.

At discharge, the patient was fully recovered without any neurological deficits, which gradually subsided following the lumbar puncture. On follow-up visit one month after she had no complaints, her neurological examination was unremarkable, and follow-up MRI scan showed complete regression of previously described lesions.

## 4. Discussion and Literature Review

We report two pediatric cases of ME caused by Mp with an unremarkable respiratory involvement and no signs of pneumonia prior or during the extrapulmonary phase of the disease. MRI in both patients demonstrated symmetrical basal ganglia involvement, with marked, predominantly mononuclear CSF pleocytosis and elevated CSF protein levels. Both patients achieved a favorable clinical and radiological recovery following initiation of non-pulse doses of corticosteroid therapy. While respiratory symptoms are often documented in patients with MPE, both of our cases involved older children who experienced only mild respiratory complaints prior to the onset of neurological symptoms. Unlike cases described so far, a favorable outcome without sequelae was observed [13,15,16]. The constellation of aforementioned symptoms, MRI and CSF findings should raise suspicion for Mp infection as a potential etiology of ME syndrome, even in the absence of respiratory symptoms, especially in the setting of older children. Given the likely immune-mediated, post-infectious pathogenesis of the disease and relatively mild clinical course, non-pulse doses of corticosteroid regimens appear to be a reasonable and safe therapeutic option, associated with rapid clinical recovery.

MPE, with or without concomitant meningitis, is the most common form of neurological manifestation of Mp infection with typical predominance among younger age groups, except for infants [8,17,18]. The condition is more frequent among pediatric patients compared to adult ones, although the exact prevalence may be underestimated due to limited diagnostic capabilities [3,19].

Among pediatric cases, clinical presentation of MPE usually includes fever, vomiting, headache, positive meningeal signs, and altered consciousness [17,18]. However, among a substantial number of pediatric patients less typical symptoms including focal neurological signs and hallucinations have been reported [17,18,20]. Preceding respiratory symptoms occur in more than 80% of patients [4]. The mean time interval between the onset of respiratory symptoms and CNS manifestations ranges between 2 and 14 days, although respiratory symptoms prior to the extrapulmonary disease can be nonspecific and minimal, presenting with rhinitis or pharyngitis, as seen in our cases [3,6]. On the contrary, the prevalence of associated respiratory symptoms during the extrapulmonary phase of the disease appears to be variable [17,18,21]. Among 87 Chinese children diagnosed with MPE almost all (94.3%) of the children experienced respiratory complaints, with confirmed concomitant pneumonia in around one-third of patients [21]. Conversely, among 84 pediatric patients with MPE included in California Encephalitis Project, only 44% experienced concomitant respiratory symptoms, and a similar proportion was found in a smaller study by Pönkä et al., which included 15 pediatric patients diagnosed with MPE [17,18]. Based on this evidence, a high index of clinical suspicion is necessary among patients presenting with meningoencephalitis or other neurological manifestations consistent with Mp infection, even in the setting of minor or absent respiratory complaints.

Mp has been associated with various other neurological manifestations, reflecting distinct pathogenetic mechanisms triggered by the agent [5]. In patients with MPE, diverse forms of the disease have been described, including mild encephalopathy with reversible splenial lesions (MERS), diffuse meningoencephalitis, striatal encephalitis, ADEM, and Bickerstaff’s brainstem encephalitis [15,22,23,24]. Due to the pathogen’s ability to cause endothelial injury, cases of acute bilateral thalamic and striatal necrosis, ischemic stroke, and hemorrhagic leukoencephalitis have also been attributed to Mp [8,14,16,25,26,27,28]. In general, neurological manifestations associated with Mp can be classified as early- or late-onset, depending on the underlying mechanism of infection, with late-onset manifestations typically attributed to immune-mediated mechanisms [6,7,9]. In case of immune-mediated disease, serum IgM antibodies are typically detected, whereas the pathogen is usually absent from CSF, probably due to immune system clearance [7]. This mechanism likely explains negative CSF PCR results in our cases. Among children hospitalized due to Mp infection, around one-fourth experienced neurological disease, with ME, aseptic meningitis, and transverse myelitis being most common clinical forms of the infection [7,26]. Less frequent manifestations include peripheral neurological disease, such as Guillain-Barré syndrome and vestibular neuritis [6,26]. The presence of genetic material in CSF obtained through PCR testing, implies that Mp has potential for direct invasion in CNS, which serves as an explanation for early-onset neurological manifestations of the infection [7,11,29]. Early-onset manifestations are usually observed among younger children, probably due to greater permeability of BBB in this group, which makes them more susceptible to direct invasion of the bacteria [11,21]. In these patients, a prodromal phase characterized by respiratory symptoms is usually absent or shorter in duration, along with a reduced IgM antibody response [7]. While the pathophysiological background of cell injury is well studied on models including epithelial cells of the respiratory tract, molecular mechanisms underlying direct neuronal damage remain unclear and warrant further studies [30,31]. Immune-mediated mechanisms are thought to play the central role in the development of neurological manifestations through molecular mimicry, immune-complex deposition, and autoantibody production [31]. Although one of our patients had a positive PCR result from a respiratory swab, there were no clinical or radiological findings of respiratory infection at the time of presentation. This can be explained by the well-documented persistence of PCR positivity in respiratory samples among children with prior RTI, which further supports the interpretation that the CNS disease in our patients was immune-mediated rather than a consequence of active infection [32,33]. In children, age does not affect the involvement and extension of extrapulmonary sites of the infection [2]. However, neurological manifestations of the disease tend to occur more often in children older than 6 years of age, with the majority of cases reported in children up to 10 years, and rarely in adolescents [8,17,18]. Simultaneously, older age is related to poorer outcome [21]. Given the rarity of extrapulmonary manifestations of Mp, including CNS disease among adults, age-related differences in immune response might be pivotal in development of these complications, highlighting the need for further research [34].

Several factors pose a challenge in diagnosing Mp-related CNS disease: the well-documented ability of Mp to cause subclinical and mild respiratory infections, particularly in children during outbreaks, the prolonged detection of genetic material in respiratory specimens and the persistence of serum IgM antibodies, and rarely observed presence of positive PCR in CSF [3,32,34,35]. As per current International Encephalitis Consortium guidelines, patients with presumed MPE, serological studies and PCR testing from respiratory tract should be routinely performed, while PCR testing of CSF samples is considered optional, although it should be performed in patients with positive results of previously aforementioned tests [36]. While the combination of serology and molecular testing improves diagnostic yield, positive results of these tests fail to reliably discriminate between colonization, past exposure, and active infection [37]. This reinforces the requirement for specific diagnostic tools in the future. Among pediatric patients with confirmed Mp infection, high rates of co-pathogens are detected, which may further complicate establishing definite etiology [37,38]. Christie and colleagues found serological or molecular evidence of co-infection in 9 children with MPE [17]. Among 9 children with serologic evidence of Mp infection or positive PCR from throat swabs, Bitnun and colleagues identified other potential etiologic agents in 3 cases [8]. Moreover, among 30 patients with only serological evidence of Mp, EV was detected in 9 patients, followed by human-herpesvirus 6, influenza virus, and *B. henselae* [8]. These observations highlight the complexity and challenges encountered in diagnosing MPE and indicate the need for cautious interpretation of microbiological findings. In our cases, PCR and serologic testing of CSF were negative, although both patients had serologic evidence of prior Mp infection from serum samples. Of note, both of them had IgM antibodies, which are reported in patients with immune-mediated MPE [7]. Given the medical history of preceding respiratory illness and exclusion of other causes by an extensive diagnostic work-up, we consider it most likely that Mp was the causative agent. However, we acknowledge that paired serum samples were not obtained at follow-up, which would give a more accurate understanding of the temporal relationship between infection and disease onset.

CSF findings in MPE are typically nonspecific and usually show mild to moderate pleocytosis with a lymphocytic predominance, and slightly elevated protein levels [39]. Conversely, our patients had a higher degree of pleocytosis than usually reported in the literature. This might reflect a more robust immune response within the CNS followed by an increased leukocyte recruitment into the CSF [40]. While the evidence of intrathecal synthesis of antibodies against Mp is highly suggestive of infection, results from clinical practice have demonstrated variable success rates of antibody detection [17,41,42]. In a smaller study by Bencina et al., intrathecal synthesis was linked with higher CSF PCR detection and pathogen isolation rates [43]. Interestingly, presented patients did not have evidence of intrathecal synthesis nor detection of the pathogen in CSF, while they simultaneously exhibited prominent cellular response. These observations imply great variations of Mp-induced immune response in CNS tissue, as well as the aberrant inflammatory response during the post-infectious phase. Future studies should investigate the relationship between serum and CSF antibody response and clinical course to properly address the underlying mechanisms contributing to disease severity.

Studies assessing the type and distribution of MRI lesions in MPE remain scarce and current insight into MRI features are primarily based on case reports [8,13,14,15,16,17,20,21,22,23,24,26,41,42,44,45,46,47,48,49,50,51,52,53,54,55,56,57]. The scarcity of MRI data might be explained by the fact that MRI is a relatively novel neuroradiological imaging tool and is not routinely used in clinical practice. The ongoing increase in MRI utilization for the diagnosis of ME is expected to yield a more comprehensive insight regarding CNS lesion distribution and significance. Studies reporting MRI lesions in MPE are presented in Table 2. A substantial number of patients diagnosed with MPE have normal MRI findings, or nonspecific diffuse abnormalities [17]. It is possible that the high proportion of normal MRI findings is partly due to the short interval between symptom onset and imaging, as certain lesions may develop later, resulting in initially false-negative results of neuroradiological assessment. In our cases, MRI was performed relatively soon after the onset of neurological symptoms. However, if the preceding respiratory illness is considered the initial point of infection, the interval becomes substantially longer. This makes the assessment of MRI lesions in the course of the illness difficult. According to the largest reported cohort of MPE patients in China, consisting of 87 pediatric cases, 25 patients had gray matter lesions, while only 6, 5 and 4 patients had experienced basal ganglia, thalamic and corpus callosum lesions, respectively [21]. MRI findings consistent with rhombencephalitis have been frequently documented as well, sometimes in conjunction with basal ganglia lesions [13,22]. Kikuchi et al. described a case of a patient with serological evidence of Mp infection and brainstem lesions, associated with high anti-GQ1b antibody titers, suggesting Mp as a trigger of Bickerstaff’s encephalitis [23]. However, isolated cases of extensive and ‘silent’ MRI lesions of the CNS have been described among adolescents, adding uncertainty regarding significance of MRI findings and highlighting the need for further research [53,58].

Basal ganglia lesions are a non-specific feature that can occur due to a variety of underlying causes, including inherited metabolic or degenerative CNS disorders, autoimmune diseases, iatrogenic causes, along with substance misuse and infectious causes [59]. Given the diversity of causes, thorough diagnostic work-up is essential to establish proper diagnosis. Diagnostic work-up should be guided by clinical judgement, medical history and other relevant information. Similar to our cases, reported MPE cases have encountered difficulties in establishing diagnosis by using currently available diagnostic tools. In most of the cases with described MRI features, diagnosis of MPE was established by serum serology alone, while changes in antibody titers were observed in only three studies [14,44,46]. Considering the limitations of serological tests, the diagnosis of MPE in most of the cases, including the ones we have described, is presumptive rather than definitive. PCR from CSF samples was positive in only 33 patients in total suggesting low diagnostic yield of molecular testing of CSF samples [8,21,41,48]. Mp detection from respiratory specimens was higher, with total of 73 patients having positive PCR results [8,17,21,47,48,55]. However, PCR testing was not routinely performed at the time some of these cases have occurred.

In total we identified 21 patients with basal ganglia involvement. All cases exhibited symmetrical lesions, suggesting underlying immune-mediated cause, although metabolic disorders can also cause this type of lesions [59]. This is particularly important in the absence of respiratory symptoms, as it should prompt the clinician to exclude other causes, primarily metabolic and autoimmune. Although basal ganglia involvement appears as a recurring phenomenon in MPE, it is sometimes accompanied with lesions of other CNS regions including thalami, cortex, and pons [15,46,52,53,56]. Interestingly, basal ganglia involvement is mostly described feature in children younger than 10 years old [13,15,16,52,55]. The exception is the case of a 17-year-old boy with a progressive akinetic-rigid syndrome reported by Termine and colleagues [42]. Unlike previously mentioned case, it seems that described basal ganglia lesions in our patients have no clinical repercussions, since they did not experience symptoms associated with basal ganglia dysfunction. Nonetheless, these lesions might serve as radiological marker of the condition, underscoring the need for proper monitoring and recognition of possible similar cases in the future. The exact mechanism underlying susceptibility of neurons of basal ganglia during the infection is still unknown, although observed mechanisms causing neuronal damage in infection-related movement disorders have been extensively studied [60].

Among 58 patients diagnosed with MPE reported by Daxboeck and colleagues, favorable recovery occurred in around half of cases, while around one-third experienced sequelae, although the impact of MRI lesions on the outcome has not been studied [39]. In most of the cases of infection-related movement disorders, deviant immune response is self-limiting, with good outcomes observed following initiation of steroid therapy [60]. The contribution of basal ganglia lesions on outcomes of patients diagnosed with MPE has not been completely elucidated, mostly due to the limited number of cases available and variable time of follow-up [8,13,14,15,21,26,42,45,46,49,52,55,56]. In isolated case reports and case series, reported outcomes are heterogeneous. Most commonly reported sequelae included persistent dystonia, chorea, muscle weakness, although cases of ataxia, muscle spasticity, cognitive decline, concentration difficulties, and obsessive-compulsive disorder have also been noted [8,13,14,15,16,17,22,26,42,45,46,49,52,53,55]. Fan and colleagues argue that basal ganglia lesions have been associated with poorer outcomes when compared to other MRI findings [21]. In our cases, clinical and MRI follow-up scans revealed favorable radiological outcomes with significant lesion regression, with no novel symptoms or complaints. However, a case described by Donovan and Lenn reported a delayed onset of chorea and basal ganglia lesions on MRI one year after initial recovery from meningoencephalitis with early CT evidence of basal ganglia involvement [15]. In the light of the aforementioned case, a longer follow-up may be warranted for timely recognition of possible late-onset sequelae.

No definite consensus regarding treatment of MPE and other neurological manifestations related to Mp exists, although treatment usually includes the use of immune-modulating drugs, alone or with antibiotic treatment [30]. Larsen and Crisp reported a case of serologically confirmed MPE with recovery achieved with supportive treatment alone, supporting the evidence that post-infectious complications might be self-limited [14]. The justification for treatment strategies mostly relies on the hypothesized pathophysiological mechanisms of the infection, which include direct bacterial invasion and damage produced by cytokine response or indirect, mediated by autoimmune reaction triggered by the pathogen [5,30,31]. In the case of early-onset neurological disease, antimicrobial therapy might be beneficial, since it could help eradicate the pathogen and hamper further tissue damage [7,30].

By now, macrolides are frequently reported as agents for the treatment of Mp, but reconsideration of antimicrobial therapy based on geographical area is needed given the increasing prevalence of macrolide-resistant Mp strains [7,13,15,16,21,26,41,42,44,45,48,49,50,51,54,55,57,61]. Corticosteroids seem to be safe and feasible adjuvant therapeutic option in patients with MPE, albeit their usage is not universal and is determined on a case-by-case basis, including variability in dosing, which may range from low-dose regimens to high-dose pulse therapy [8,16,21,22,24,26,44,46,49,52,53,54,55,56,57]. IVIg is less commonly reported immunomodulatory agent in the treatment of MPE and is mostly used in cases with more severe clinical form [13,21,22,26,41,50,51,52]. Among patients who received antimicrobial therapy along with immunomodulatory therapy, shorter lengths of stay and better symptom control were observed [21]. To conclude, comprehensive studies, including meta-analyses are needed to properly address the impact of different treatment modalities in order to maximize patient outcomes.

## 5. Conclusions

This report highlights an unusual clinical presentation of relatively mild MPE occurring in older children, with no significant respiratory involvement, distinctive MRI features and with quick and favorable outcome following non-pulse doses of corticosteroid treatment. This particular combination of clinical, laboratory and neuroradiological features should raise clinical suspicion for Mp infection as a potential etiology of ME.

This review is the first to comprehensively investigate and to summarize neuroradiologic findings in MPE patients. Described cases contribute to the growing body of literature regarding basal ganglia lesions in MPE, suggesting it may be a neuroradiologic pattern worth recognizing as it could help guide diagnostic consideration of MPE in the absence of respiratory symptoms. However, observed variations in diagnostic approaches and occurrence of basal ganglia lesions along with other MRI abnormalities in reported studies, urge for cautious interpretation of such findings in MPE. Given the diversity in clinical presentation and diagnostic challenges of this condition, further research is needed in the future to potentially establish definite diagnostic tools and criteria to guide clinicians.

## Figures and Tables

**Table 1:** Demographic characteristics, laboratory findings, imaging results, treatment, and outcomes in patients.

	Sex	Age	CSF				MRI Findings	Treatment	Outcome
			WBC [n/µL]	PMN [%]	MNC [%]	Proteins [g/L]			
Patient 1	M	11 years and 9 months	331	44	56	0.78	T2-weighted and FLAIR images demonstrate bilaterally asymmetrical high signal intensity in the caudate nuclei, ventral portions of the globus pallidus, and putamina. A linear area of increased signal extends toward the right half of the hypothalamus. A Chiari I malformation is also noted.	Methylprednisolone (doses of 2.5 mg/kg/day for 5 days, followed by taper-off)	Full recovery
Patient 2	F	14 years and 2 months	480	15	85	1.21	T2-weighted and FLAIR images demonstrate bilaterally symmetrical high signal intensity in the thalami (pulvinar nuclei), caudate nuclei, and putamina, findings consistent with encephalitis.	Methylprednisolone (doses of 2.5 mg/kg/day for 5 days, followed by taper-off)	Full recovery

CSF—cerebrospinal fluid; F—female; M—male; MNC—mononuclear cells; MRI—magnetic resonance imaging; PMN—polymorphonuclear leukocytes; WBC—white blood cells.

**Table 2:** Studies and case reports of MPE with performed MRI among pediatric patients.

Study	No. of Patients/Age	MRI Findings	CSF Findings (WBC Count and Protein Levels)	*M. pneumoniae* Detection Techniques	Treatment
Donovan and Lenn, 1989 [15]	1 pt with MPE/7 yrs	Bilateral lesions of the putamen and basis pontes	WBCs 8/μL, protein levels 0.28 g/L	Positive *M. pneumoniae* serology (serum, high titer)	Erythromycin
Koskiniemi, 1993 [20]	45 pts/NA (mostly <5 yrs)	1/3 of pts had MRI/CT scans performed, mostly unremarkable	60% had normal WBC count and protein levels	Positive *M. pneumoniae* serology (serum)	N/A
Thomas et al., 1993 [46]	12 pts with MPE/between 2–15 yrs	6 pts had MRI scans, of which 2 had altered signal of the deep white matter	WBCs ranged from 0–110/μL, protein levels ranged from 0.11–1.5 g/L	Positive *M. pneumoniae* serology (serum—fourfold rise in antibody titer)	8 pts received erythromycin; 2 pts received corticosteroids
Brandel et al., 1996 [47]	1 pt with postinfectious MPE/5 yrs	Bilateral lesions of striata and external pallidum	WBCs 20/μL, protein levels normal	Serum—positive IgG (1:640) serology for *M. pneumoniae*	Erythromycin
Larsen and Crisp, 1996 [13]	1 pt with MPE/7 yrs	Marked hypodense lesions of the striatum	Normal WBC and protein levels	Changes in IgM and IgG (serum) *M. pneumoniae* titers	Recovered without specific treatment
Yamamoto et al., 1996 [48]	1 pt with ADEM triggered by *M. pneumoniae*/8 yrs	Findings consistent with ADEM—bilateral basal ganglia, thalami, white matter	WBCs 76/μL, protein levels normal	Positive *M. pneumoniae* serology (changes in IgM and IgG titers)	Corticosteroids
Kikuchi et al., 1997 [24]	1 pt with Bickerstaff’s brainstem encephalitis triggered by *M. pneumoniae*/7 yrs	Periaqueductal lesions	WBCs 8/μL, protein levels normal	Positive *M. pneumoniae* serology, along with high IgG anti-GQ1b titer (1:12,800)	Minocycline with plasmapheresis
Kolski et al., 1998 [49]	9 pts with MPE/1–17 yrs (mostly >5 yrs)	2 pts had diffuse edema with temporal, occipital or hippocampal involvement, 2 pts had multifocal edema or infarction, 5 pts had unremarkable scans	6 had pleocytosis (0–110 WBCs/μL), 4 had elevated protein levels (0.46–0.53 g/L)	Positive *M. pneumoniae* serology (serum) in all pts, 1 pt had PCR + from throat swab	N/A
Ieven et al., 1998 [50]	1 pt with MPE/17 yrs	Normal	WBCs 34/μL, protein levels 0.52 g/L	*M. pneumoniae* PCR + from CSF and respiratory tract	Erythromycin
Smith et al., 2000 [51]	4 pts with MPE/5–11 yrs	Bilaterally increased signals of caudate nucleus, globus pallidum, internal capsule; increased signals of the left cerebellar hemisphere and the right occipital lobe	WBCs ranged 0–45/μL, CSF protein levels ranged 0.23–0.3 g/L	Positive *M. pneumoniae* serology (serum)	3 and 2 pts received corticosteroids and macrolides, respectively
Bitnun et al., 2001 [7]	20 pts with MPE	6 pts had MRI: 3 had focal edema or ischemia, 1 had edema of the basal ganglia, 1 had focal enhancing lesion, 1 had lesions consistent with ADEM	11 pts had pleocytosis (WBCs 6–110/μL), 8 had elevated CSF protein levels (0.1–1.19 g/L)	6 pts had PCR+ in CSF, 6 pts had PCR + from throat swabs, 14 pts had IgM + in acute-phase sera	Corticosteroids, macrolides, doxycycline
Riedel et al., 2001 [43]	1 pt with ADEM triggered with *M. pneumoniae*/17 yrs	Hyperintense lesions of the right parietal lobe and corpus callosum, perifocal edema of white matter, lesions of posterior crus of internal capsule and dorsal hippocampus, lesions of the right cerebellar hemisphere	WBCs 2900/μL, protein levels normal	Positive *M. pneumoniae* antibodies detected in both serum and CSF, *M. pneumoniae* PCR + in CSF	Erythromycin + IVIg
Sakoulas, 2001 [12]	1 pt with MPE (brainstem encephalitis)/6 yrs	High signal lesions of pontine-medullary junction of the brainstem, along with bilateral caudate and putamen lesions	Normal WBC and protein levels	Positive serology (serum); *M. pneumoniae* PCR + from throat swab	Azithromycin, IVIg
Ashtekar et al., 2003 [16]	1 pt with MPE/5 yrs	Acute bilateral thalamic necrosis	Not performed due to severe clinical form	Positive *M. pneumoniae* serology (serum)	Erythromycin, pulse doses of methylprednisolone
Tan et al., 2003 [52]	1 pt with MPE and Guillian-Barré syndrome/7 yrs	Extensive multifocal, confluent high signal lesions involving deep and superficial white matter in both hemispheres, along with reduction of brain volume and volume of both cerebellar hemispheres	Normal WBC and protein levels	Positive *M. pneumoniae* serology (serum); raised anti-ganglioside GM1 titer (1:1090)	Erythromycin + IVIg
Termine et al., 2005 [44]	1 pt with MPE/17 yrs	Cytotoxic edema restricted specifically to both the striata	WBCs 11/μL, normal protein levels	Positive *M. pneumoniae* serology (IgM and IgG) in serum and CSF	Erythromycin
Christie et al., 2007 [17]	84 pts with MPE/median 11 yrs	27 pts had normal MRI; 44 pts had abnormal MRI findings, of which 13 had diffuse lobar changes, 9 had white matter lesions, 6 had lesions of thalami, 3 had brainstem changes, 5 had gray matter or single lobe abnormalities	WBCs ranged 0–990/μL, protein levels ranged 0.07–3.6 mg/dL	Positive *M. pneumoniae* serology (serum); 3 pts had positive CSF serology PCR + from respiratory samples 5	N/A
Yiş et al., 2008 [53]	2 pts with MPE, one of which had lesions consistent with ADEM/10 yrs (both pts)	One MRI showed lesions consistent with ADEM (brainstem, cortical white matter), the other was normal	Mild pleocytosis (WBCs 10–20/μL), normal protein levels	Positive *M. pneumoniae* serology (serum) in both patients	Clarithromycin in both pts (+ IVIg in pt with ADEM
Fusco et al., 2010 [54]	1 pt with MPE/2 yrs	T2-hyperintensities in basal ganglia and thalami	WBC normal, protein levels of 0.88 g/L	Positive *M. pneumoniae* serology (serum), along with positive anti-GM1 antibodies titer (1:300)	Methylprednisolone (pulse doses) + IVIg
Bae et al., 2011 [55]	1 pt with optic neuritis and ‘silent’ brain lesions/7 yrs	“Silent” extensive symmetrical lesions involving striatum, midbrain, pontine tegmentum, and right subcortical cerebellar white matter, along with optic nerve lesions	Normal WBC and protein levels	Positive *M. pneumoniae* serology (serum)	Corticosteroids
de Lalibera et al., 2012 [56]	1 pt with MPE/5 yrs	Rhombencephalitis (unspecific signal changes in the pons)	WBCs 350/μL, elevated protein levels 0.55 g/L	Positive *M. pneumoniae* serology (serum)	Clarithromycin, prednisone
Erol et al., 2013 [23]	2 pts with ADEM triggered by *M. pneumoniae*/0.5 and 8 yrs	Lesions consistent with ADEM, the first pt had thalamic lesions and brainstem involvement, other pt had frontal, temporal, parietal and subcortical lesions	1 pt had CSF pleocytosis (not specified), protein levels were 0.23 and 0.44 g/L	Positive *M. pneumoniae* serology (serum)	Clarithromycin, pulse doses of methylprednisolone, IVIg in one pt
Yuan et al., 2016 [57]	1 pt with MPE/8 yrs	Bilateral striatal lesions	Normal CSF WBC and protein levels	Positive *M. pneumoniae* serology (serum), PCR + from respiratory specimen	Azithromycin, pulse doses of methylprednisolone
Kammer et al., 2016 [28]	6 pts with MPE/between 5–13 yrs	2 pts with normal MRI, 4 patients with findings in supratentorial white matter, infratentorial gray matter, supratentorial cortex, leptomeningeal enhancement, and 1 pt had basal ganglia involvement	3 pts had pleocytosis—WBC between 9–84/μL; 5 pts with normal CSF protein levels, 1 pt 0.61 g/L	Positive *M. pneumoniae* serology (serum)	Macrolide in 5 pts, prednisolone in 4 pts, IVIg in 1 pt
Ueda et al., 2016 [26]	2 pts with MPE/mild encephalopathy/14 and 8 yrs	Both patients had hyperintensities of the splenium of CC, one additionally had lesions of the left cerebellar hemisphere	N/A	Positive *M. pneumoniae* serology (serum)	Minocycline in both pts, dexamethasone in 1 pt
Smolders et al., 2019 [58]	1 pt with MPE/9 yrs	T2/FLAIR hyperintense signals of basal ganglia and cortex	WBCs 166/μL, normal protein levels	Positive *M. pneumoniae* serology (serum); positive IgM and IgG antiganglioside antibodies	High-dose methylprednisolone
Fan et al., 2022 [22]	87 pts with MPE/median 5 yrs	Lesions in gray matter, corpus callosum, brainstem, thalamus, cerebellum; 6 pts had basal ganglia lesions	Mild pleocytosis (WBC median 182/μL), 55% had elevated protein levels	Positive *M. pneumoniae* serology (serum) in 70 pts; 25 pts had PCR + from CSF sample; 58 pts had PCR + from throat swab	Azithromycin ± IVIg or steroids (methylprednisolone low and high doses)
Khan et al., 2022 [59]	1 pt with MPE/11 yrs	Rhombencephalitis	WBCs 85/μL, protein level 0.9 g/L	Positive *M. pneumoniae* serology (serum)	Azithromycin, methylprednisolone

ADEM—Acute Disseminated Encephalomyelitis; CSF—Cerebrospinal Fluid; CT—Computerized Tomography; IVIg—Intravenous Immunoglobulins; MPE—*M. pneumoniae* encephalitis; MRI—Magnetic Resonance Imaging; N/A—Not available; PCR—Polymerase Chain Reaction; Pt(s)—patient(s); WBC—White Blood Cell; yr(s)—year(s).

## Data Availability

The original contributions presented in this study are included in the article. Further inquiries can be directed to the corresponding author(s).

## Abbreviations

ADEM	Acute Disseminated Encephalomyelitis
BBB	Blood-Brain Barrier
CNS	Central Nervous System
CSF	Cerebrospinal Fluid
EV	Enterovirus
HSV	Herpes-simplex Virus
LP	Lumbar Puncture
ME	Meningoencephalitis
MERS	Encephalitis with a Reversible Splenial Lesions
Mp	*Mycoplasma pneumoniae*
MPE	Mycoplasma pneumoniae-associated encephalitis
MRI	Magnetic Resonance Imaging
PCR	Polymerase Chain Reaction
RTIs	Respiratory Tract Infections
VZV	Varicella-zoster Virus
